# Somapacitan-induced reversible lipoatrophy in an adult woman with hypopituitarism

**DOI:** 10.1007/s11102-024-01440-w

**Published:** 2024-08-09

**Authors:** Matej Rakusa, Andrej Janez, Mojca Jensterle

**Affiliations:** Faculty of Medicine, University Medical Centre Ljubljana, University of Ljubljana, Zaloška cesta 7, Ljubljana, SI-1000 Slovenia

**Keywords:** Lipoatrophy, Somapacitan, Long-acting growth hormone

## Abstract

**Background:**

Lipoatrophy is rare adverse event (AE) in daily recombinant human growth hormone (rhGH). Data on lipoatrophy in newly developed long-acting GH (LAGH) are scarce. We report the first case of lipoatrophy in adult patient treated with LAGH somapacitan.

**Case presentation:**

A 38-year-old woman with congenital panhypopituitarism was transitioned from daily rhGH 0.4 mg QD to somapacitan dose 4 mg QW due to non-adherence to daily rhGH. Despite adequate education and regular changing of injection sites, the patient reported reduced subcutaneous tissue at all four injection sites, after the 4th application of somapacitan. Somapacitan was discontinued at patient preference and lipoatrophy completely reversed after 3 months.

**Conclusions:**

Lipoatrophy caused by somapacitan was completely reversible. We speculate that high initial dose and volume of somapacitan caused delayed diffusion and a direct local lipolytic effect in our patient. Although, titration of somapacitan was initiated as previously reported in REAL2 study protocol, recent clinical guidelines advise more gradual increase of somapacitan dose also in women on oral estogens that are switched from daily rhGH. Importantly, our case and the two previously described cases in children in the REAL 3 study showed that lipoatrophy caused by somapacitan was transient and completely reversible, and that discontinuation of the drug is not always mandatory.

To the Editor

As long-acting growth hormone (LAGH) formulations are finally emerging in clinical practice as a solution to address the challenges of daily subcutaneously injections of recombinant human GH (rhGH) in patients with growth hormone deficiency (GHD), we think it is important to expand the limited knowledge on the local adverse effects of the LAGH. While lipoatrophy was frequent in earlier pegylated LAGH, data in newly developed long-acting GH (LAGH) are scarce.

Somapacitan is LAGH derivate with high similarity to endogeneous GH, that achieves extended action trough non-covalent albumin binding, via attached fatty acid. It has been recently approved for the treatment of adult GHD with an established efficacy and favourable safety profile. Herein, we report the case of reversible lipoatrophy in 38-year-old woman with congenital panhypopituitarism who was switched from daily recombinant human growth hormone to somapacitan due to non-adherence to daily rhGH.

At the time of introducing somapacitan, her medication regimen included hydrocortisone (20 mg daily divided), levothyroxine (100 mcg QD), combined norgestrel/estrogen hormonal contraception (QD) and daily rhGH 0.4 mg QD. Her IGF-1 level was 72 µg/l (reference range: 69.0–227.0 µg/l), with an IGF-1 SDS of -1.825, IGFBP-3 2.24 mg/l (reference range: 2.50–5.71 mg/l), IGFBP-3 SDS − 2.298. Her kidney and liver function, fasting glucose, HbA1c and HOMA-IR were normal. Her BMI was 21.7 kg/m^2^, visceral adipose tissue (VAT), measured with dual X-ray absorptiometry (DXA) was 69.8 cm^2^.

In the last year she reported to skip approximately 20% of rhGH applications. We offered her a switch from daily rhGH to LAGH somapacitan to improve the adherence. She received comprehensive education on proper injection technique and regular site rotation. The initial dose of somapacitan was 4 mg QW, as advised in the REAL2 study protocol for switching from daily rhGH to somapacitan in patients on oral estrogens [1]. On the 3rd day after the second injection of somapacitan (the 10th day after initiation of treatment), her serum IGF-1 increased to 140 µg/l (IGF-1 SDS: 0.143). After the 4th application of somapacitan she called us and reported reduced subcutaneous tissue at all four injection sites. Examination confirmed loss of subdermal adipose tissue on the lateral thighs and left and right side of lower abdomen, with absent pain, redness and excess warmth of the skin (Fig. 1A). IGF-1 was within targeted range 148 µg/l (IGF-1 SDS: 0.323). Marginal changes in local fat loss have been detected by DXA (left leg: mass from 3559 g to 3484 g, fat percentage from 36.6 to 35.7%; right leg: mass from 3944 g to 3652 g, fat percentage from 40.8 to 39.1%). Bioactive leptin (18.16 µg/l) and total leptin (22.53 µg/l) levels were also within normal ranges. Anti-somapacitan and anti-GH antibody testing was unavailable. Despite improved quality of life as assessed by SF-36v2 questionnaire and normalized IGF-1 SDS, somapacitan was discontinued due to patient preference. The patient was transitioned back to daily rhGH therapy. Follow-up visits documented partial improvement of lipoatrophy at 1 month and complete resolution by 3 months after somapacitan cessation (Fig. 1B). Two other cases of lipoatrophy were reported in two children treated with somapacitan 0.04 mg/kg/week [2]. Both recovered after change of injection site, with no dose adjustment necessary [2].

We speculate that high initial dose and volume of somapacitan caused delayed diffusion and a direct local lipolytic effect in our patient. The latest clinical recommendations suggest a more gradual increase in the dose of somapacitan, starting with 2 mg QW even in women taking oral oestrogens who have switched from daily rhGH [3]. Moreover, when high doses of long-acting growth hormone analogue (LAGH) are required in women treated with oral estrogens, a switch from oral to transdermal oestrogen may be considered to obtain optimal IGF-1 at lower dosage of LAGH and potentially reduce the risk for local side effects.

An important take-home message from our case of somapacitan-induced lipoatrophy, which is the first reported in an adult patient, and from the two previously described cases in children in the REAL 3 study [2], is that lipoatrophy caused by somapacitan is transient and completely reversable and that discontinuation of the drug is not mandatory.

**Fig. 1:**
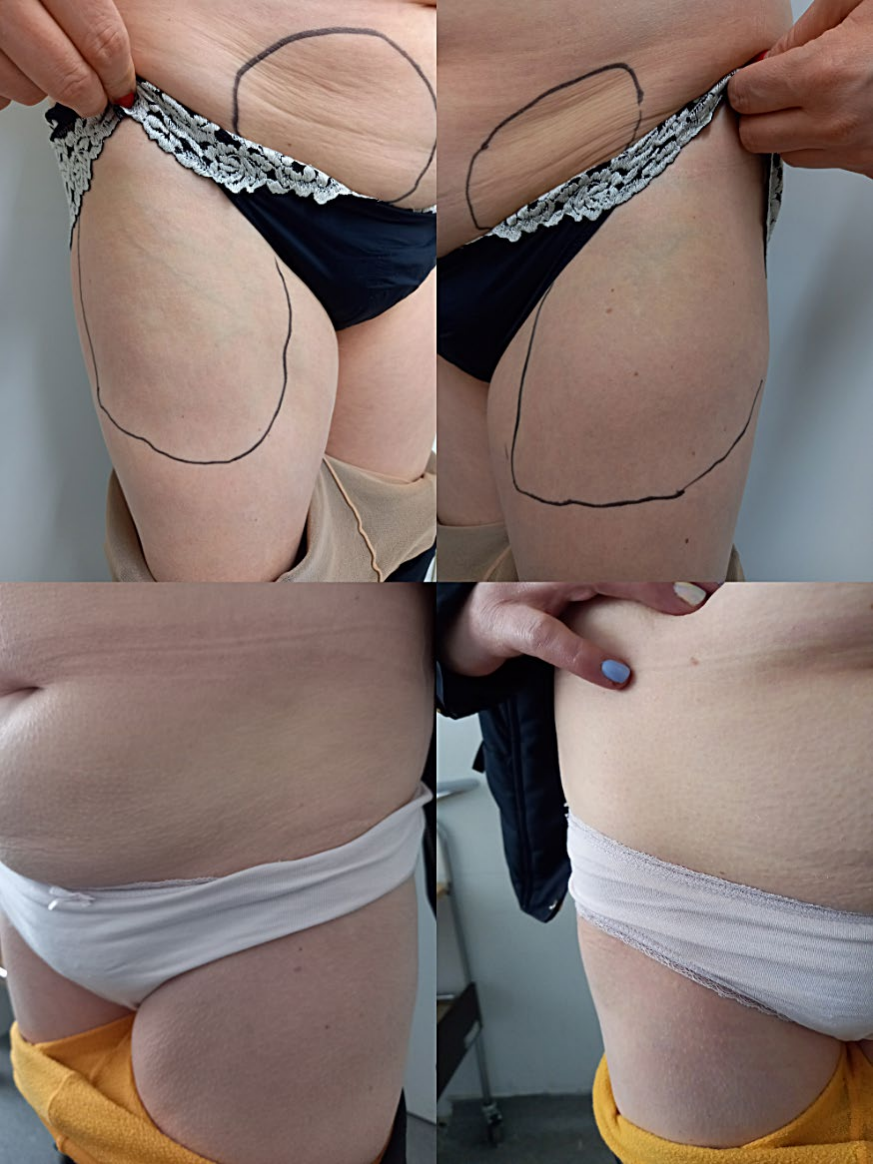
**A** Lipoatrophy of the lateral thighs (left: 10 × 7 cm, right: 11 × 6.5 cm) and lower abdomen (left side: 15 × 7.5 cm, right side: 14 × 7 cm), 4 weeks post somapacitan initiation. **B** Intact skin and subdermal tissue approximately 3 months post somapacitan discontinuation

## Data Availability

No datasets were generated or analysed during the current study.
